# Effects of a Short Heat Treatment Period on the Pullout Resistance of Shape Memory Alloy Fibers in Mortar

**DOI:** 10.3390/ma12142278

**Published:** 2019-07-16

**Authors:** Min Kyoung Kim, Dong Joo Kim, Young-Soo Chung, Eunsoo Choi

**Affiliations:** 1 Department of Civil and Environmental Engineering, Sejong University, 2019, Neungdong-ro, Gwangjin-Gu, Seoul 05006, Korea; 2 Department of Civil Engineering, Chung-Ang University, 221 Heuksuk-dong, Dongjak-gu, Seoul 156-756, Korea; 3 Department of Civil Engineering, Hongik University, 72-1 Sangsu-dong, Mapo, Seoul 121-791, Korea

**Keywords:** fibers, smart materials, fiber/matrix bond, physical properties, heat treatment

## Abstract

The feasibility of the crack closure of cementitious composites reinforced with shape memory alloy (SMA) fibers was investigated by performing single-fiber pullout tests. To demonstrate the fast crack closing ability, in this study, a heat treatment (300 °C) was applied for a short time (10 min). A short heat treatment was applied for 10 min, after the slip reached 0.5 mm, to activate the shape memory effects of cold-drawn SMA fibers. Two types of alloys were investigated, NiTi and NiTiNb, with two geometries, either smooth or dog-bone-shaped. During the heat treatment, the pullout stress of the SMA fibers initially decreased due to thermal extension, and then increased after heating for 1–3 min, resulting from the shape memory effects. However, their pullout stress recovery during and after the heat treatment was different for the different alloys and fiber geometries. The NiTi fibers generally produced a higher and faster recovery in terms of their pullout stress than the NiTiNb fibers, while the dog-bone-shaped fibers showed a faster pullout stress recovery than the smooth fibers.

## 1. Introduction

Considerable research has been performed on extending the service life of civil infrastructures through the prevention of early concrete deterioration. As an early deterioration countermeasure, various high-performance construction materials, such as ultra-high-performance concretes (UHPCs), high-performance fiber-reinforced cementitious composites (HPFRCCs), and self-healing concretes (SHCs), have been developed by numerous researchers [1,2,3,4,5,6]. However, even the high-performance construction materials are unable to close existing concrete cracks in a short time for a quick (or instantaneous) repair of concrete infrastructure. Although both the UHPCs and HPFRCCs have been shown to produce a significantly high tensile strength and cracking control capacity by generating multiple microcracks during tensile strain hardening [1,7], as well as a larger redistribution capacity of stresses [8], they remain unable to close existing cracks. Although the SHCs can heal (or fill) cracks by precipitating crystalline calcium carbonates within the cracks, they require a water supply and a minimum of a few days to heal the cracks [5,9]. Therefore, the aforementioned high-performance construction materials cannot be applied for urgent repairs, even though they have shown a superior prevention of early concrete deterioration.

Due to the issues noted above, the authors of this study propose the development of short shape memory alloy (SMA) fiber-reinforced cement composites with a fast crack closing capacity. Kim et al. [10,11] have already demonstrated that an 8 h long-term heat treatment of cold-drawn SMA fibers significantly enhanced the pullout resistance of fibers embedded in a mortar matrix due to the diameter recovery in the cold-drawn SMA fibers. However, it is quite difficult to maintain a heat treatment for a long time on site. Kim et al. [12] demonstrated the pre-stressing effect of shape memory alloy fiber-reinforced cementitious composites (SMA-FRCCs) under direct tension after a short (10 min) heat treatment. They reported that the shape memory effect can be activated by short-term heat treatment for 10 min. Consequently, in this study, we investigated whether a short (10 min) heat treatment period could activate the shape memory effect and eventually close existing cracks. 

This research is aimed at developing SMA-FRCCs with a crack closing capacity, as well as pre-stressing effects by utilizing the shape memory effect. The detailed objectives are (1) to investigate the geometry change in the SMA fibers during short heating periods, (2) to investigate the heat treatment effects on the pullout resistance of the SMA fibers, and (3) to discover the influence of different SMA fiber geometries and compositions on the pullout resistance recovery during and after the short heat treatment period.

### Shape Memory Alloy Fiber-Reinforced Cementitious Composites (SMA-FRCCs)

Considerable research has been performed on the application of SMA wires or bars to civil infrastructures and buildings by activating their shape memory effects and utilizing their super-elastic characteristics. Researchers have reported on the effectiveness of wrapping SMA wires around concrete cylinders or columns to enhance ductility and strength by utilizing the confinement effects induced from shape memory effects. Choi et al. [13] proposed a new jacketing method to confine concrete cylinders and/or reinforced concrete columns using SMA wires, demonstrating that the shape memory effect of SMA wires was considerably more effective than the steel jacketing method for generating confinement effects [14,15]. Tran et al. [16] also reported that the shape memory-induced confinement effect of SMA wires significantly increased the strength and ductility of concrete cylinder samples. Furthermore, SMA wires have also been used to reinforce cement-based matrices in order to generate pre-stressing effects in the cement composites; Sawaguchi et al. [17] applied Fe-Mn-Si-based shape memory alloys containing NbC to generate pre-stressing effects.

In addition to the confinement and pre-stressing effects obtained through SMA wire application, the self-crack closing behavior of composites reinforced with SMA wire has also been investigated. SMA wires added to epoxy or cement mortar were applied to generate a crack closing capability in a beam [18,19,20]. In order to create the crack closing ability, it is important to have a strong interfacial bond strength between the fiber or wire and the surrounding matrix. Accordingly, Umezaki [18] investigated the pullout resistance of SMA wires, reporting that spiral SMA wires produced a higher pullout resistance than smooth SMA wires. Wang et al. [21] investigated the internal stress distribution to prevent debonding at the SMA fiber and epoxy matrix interface, in order to eventually utilize the shape memory effect more efficiently. Watanabe et al. [22] also reported that the interfacial bond strength of Fe-based SMA wire was notably enhanced as the amount of pre-strain in the SMA wires increased. Kuang and Ou [23] developed a self-healing concrete beam by utilizing the super-elastic behavior of SMAs and the cohering characteristic of the repairing adhesive.

Although several researchers have studied the behavior of SMA wire-reinforced composites in an attempt to utilize their shape memory effect, the majority of their SMA applications have been limited to continuous SMA wires rather than short SMA fibers. The implementation of short SMA fibers has rarely been attempted, and the behavior of short SMA fiber-reinforced cementitious composites is yet to be discovered. In this study, to develop short SMA fiber-reinforced cementitious composites with a self-crack closing ability, the pullout resistance of these fibers embedded in a mortar matrix was systematically investigated by applying a short heat treatment period.

## 2. Experiments

An experimental program was designed to investigate the feasibility of crack closure of SMA-FRCCs by applying a short heat treatment period (10 min). The pullout stress recovery of SMA fibers embedded in a mortar matrix was investigated during this heat treatment with single-fiber pullout tests. Two types of SMAs (NiTi and NiTiNb) were used, while two types of fiber geometries (smooth and dog-bone-shaped) were investigated experimentally. First, the geometric changes in the diameter and length of cold-drawn SMA fibers during the short heat treatment time were investigated by analyzing stereoscopic microscope images taken every minute of the heat treatment. Second, the pullout stress versus slip (and time) responses of the SMA fibers in a mortar matrix were investigated by performing single-fiber pullout tests. The conditions of the single-fiber pullout tests are provided in Table 1. During the pullout tests, a short heat treatment period was applied while maintaining a constant slip (0.5 mm) for 10 min once the fiber slip reached 0.5 mm. Then, the fiber was pulled further until complete fiber pullout was achieved.

### 2.1. Materials and Sample Preparation

**Figure 1:**
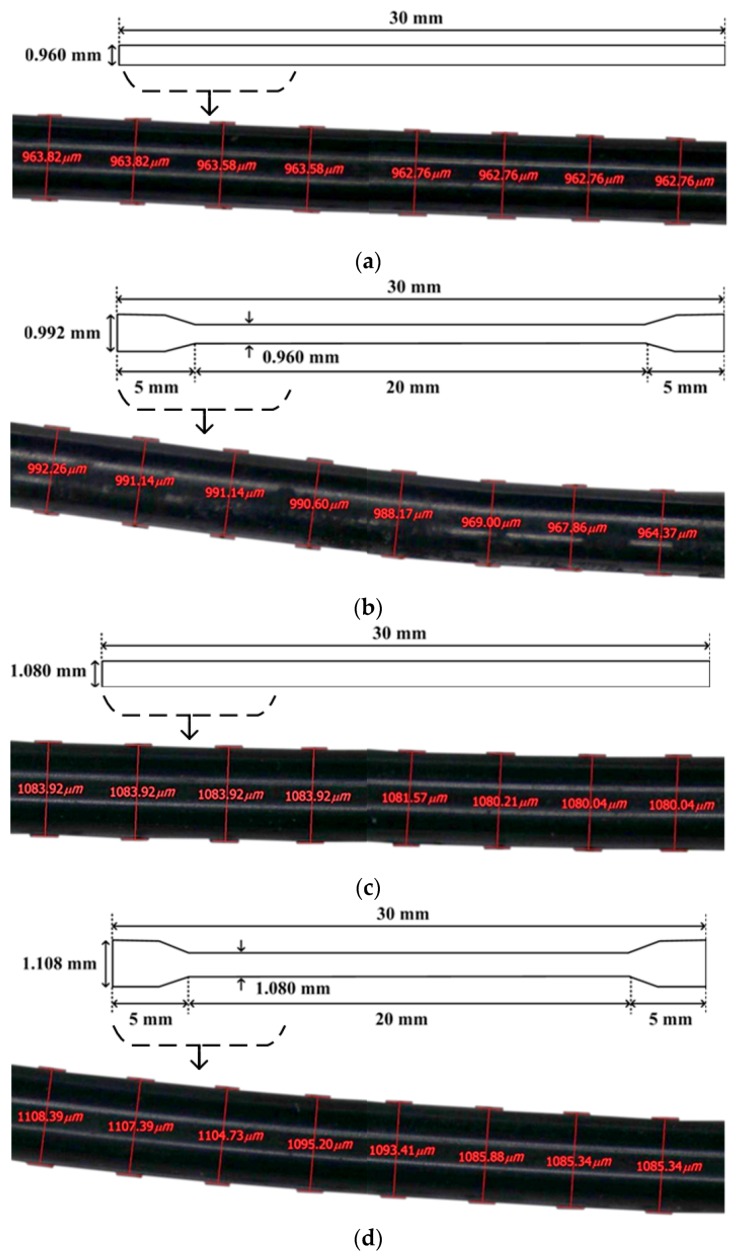
Shape memory alloy (SMA) fiber geometries, for (**a**) NiTi smooth (NT_S), (**b**) NiTi dog-bone-shaped (NT_D), (**c**) NiTiNb smooth (NTN_S), and (**d**) NiTiNb dog-bone-shaped (NTN_D).

The composition and strength of the mortar matrix are provided in Table 2; the compressive strength of the mortar was 55 MPa. The properties of the SMA fibers investigated in this study are summarized in Table 3. Four types of SMA fibers (NT_S, NT_D, NTN_S, and NTN_D, described in Table 1) were used and are shown in Figure 1. The NiTi (NT) alloy contained 50% nickel by atomic composition, whereas the NiTiNb (NTN) alloy contained 41% nickel, 50% titanium, and 9% niobium. The initial 1.0 mm diameter of the NT wires was reduced to 0.96 mm after the cold drawing process.

In comparison, the NTN wires with a 1.12 mm initial diameter were also cold-drawn to a 1.08 mm reduced diameter. The transformation temperatures of both cold-drawn NT and NTN fibers are summarized in Table 3.

Six samples of each type of SMA fiber were prepared, with three samples pulled out with the heat treatment, whereas the others were tested with no heat treatment. The notation NT_S_N in Table 1 represents the pullout samples of the NT smooth (S) fiber with no (N) heat treatment, while samples of the NTN dog-bone-shaped (D) fiber with the short heat (H) treatment periods were designated as NTN_D_H. The fibers were first installed in fiber holding devices to maintain the embedment length (15 mm) and inclination angle (90°) of the fiber within a mortar matrix. The devices holding the SMA fibers were then placed in molds to produce bell-shaped pullout samples. The mortar matrix was prepared using a Hobart-type laboratory mixer with a 20 L capacity. A detailed procedure on mixing and curing this type of sample can be found in Kim et al. [10,11]. The pullout samples, after curing in water for 14 days, were dried and tested after 16 days at room temperature in the laboratory.

### 2.2. Test Setup and Procedure

**Figure 2:**
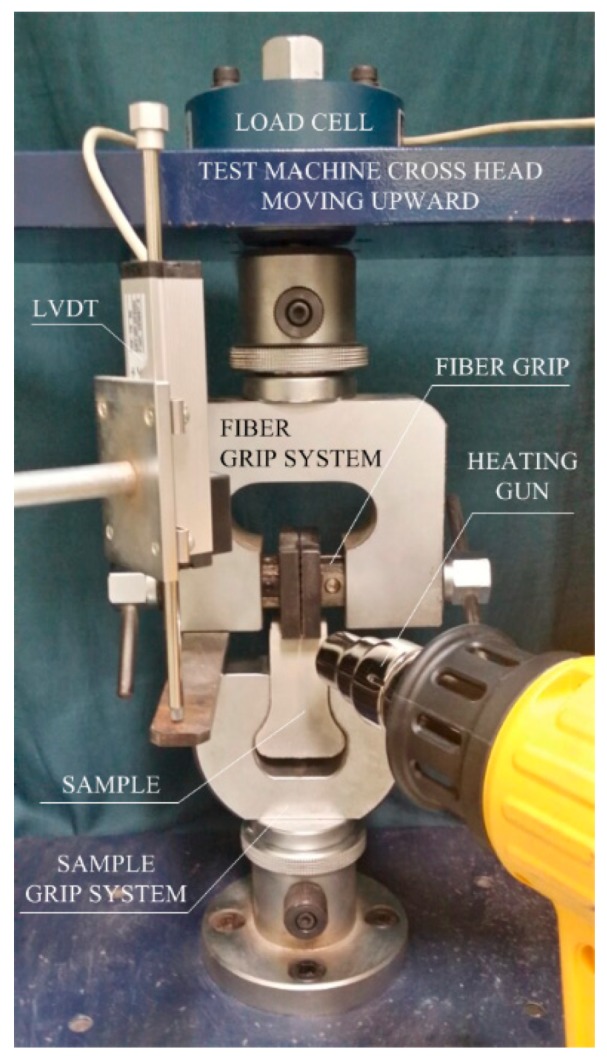
Test setup for the single-fiber pullout test.

The length and diameter of the SMA fibers could be measured by analyzing images taken by a stereoscopic microscope with a Huvitz Lusis HC-30MU camera (Huvitz, Gyeonggi-do Province, Korea). A short period of heat treatment was applied using a heat gun (DeWalt D26411) (DeWalt, Towson, MD, USA). The operation temperature of the heat gun was set at 300 °C and the airflow speed was 250 L/min. The heating gun was installed at a 35 mm distance from the SMA fibers during the heat treatment. The heat treatment in the experiment was performed using a commercial heating gun for the practical application, even in structural members. The temperature applied by using a heat gun was about 300 °C, so that it would not generate any significant damage on the cement-based matrix [24]. Therefore, it was applied to raise the temperature of SMA fibers (over A_f_) embedded within the mortar matrix. Moreover, heat treatment at 300 °C for 10 min does not significantly affect the tensile strength of SMA-FRCCs (reduced about 10%) [12], unlike SFRCs, which exhibit about a 40% reduction in tensile strength by heating at 300 °C for 2 h prior to testing [25]. During the pullout resistance investigation of the SMA fibers, a universal test machine with a 500 kgf capacity was used and the displacement speed was maintained at 1 mm/min during the fiber pullout. To avoid any slip of fiber in the grip system, the other part of embedded fiber was fully held with a sufficient pressure in the grip, as can be seen in Figure 2. The test procedure is as follows: the fiber was initially pulled out to a 0.5 mm slip with a velocity of 1 mm/min; then, the 0.5 mm slip was kept constant for 10 min; and finally, the fiber was further pulled with the same 1 mm/min velocity until complete pullout was achieved. The test set-up for the single-fiber pullout test is shown in Figure 2.

## 3. Results and Discussion

### 3.1. Length and Diameter of Cold-Drawn SMA Fibers during the Short Heat Treatment Period

**Figure 3:**
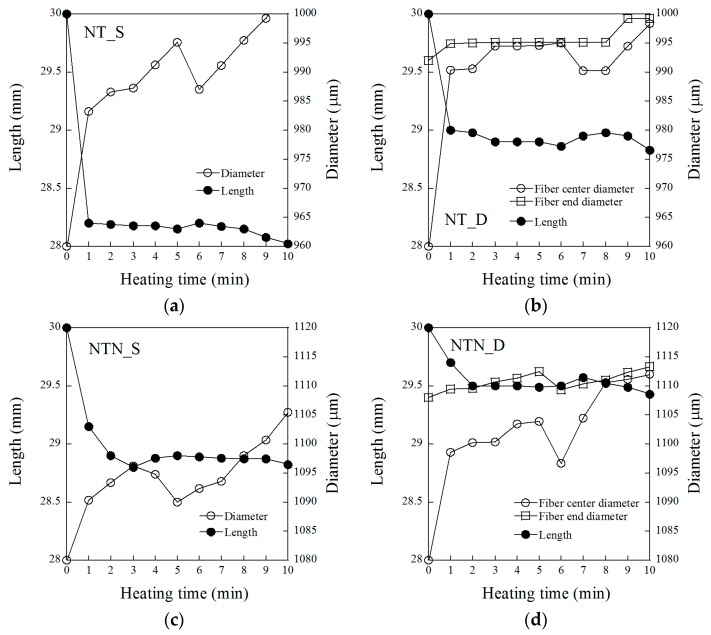
Effects of heating time on the length and diameter of the cold-drawn shape memory alloy (SMA) fibers for (**a**) NT_S, (**b**) NT_D, (**c**) NTN_S, and (**d**) NTN_D.

The changes in the diameter and length of the cold-drawn SMA fibers during the short heat treatment time are provided in Figure 3; Figure 3a–d shows the changes in the length and diameter of the NT_S, NT_D, NTN_S, and NTN_D samples, respectively. During the 10 min heat treatment, the lengths of the cold-drawn SMA fibers clearly decreased, whereas their diameters, which were originally reduced by the cold-drawing process, increased due to the shape memory effect.

Although all SMA fibers had noticeable changes in their diameter and length, the changes were different, based on the type of alloy and geometry. During the heat treatment, the NT_S length was shortened from 30 to 28 mm (−6.67%), while that of the NTN_S decreased from 30 to 28.8 mm (−4.0%), as shown in Figure 3a,c, respectively. On the other hand, the NT_S diameter expanded from 960 to 1000 μm (+4.17%), while the NTN_S increased from 1080 to 1105 μm (+2.31%). In addition, the NT_D also presented a greater length reduction (from 30 to 28.8 mm) and wider expansion of the diameter (from 960 to 998 μm) than the NTN_D, as shown in Figure 3b,d. Therefore, the NT series generally showed higher shape memory effects, for both the diameter expansion and length reduction, than the NTN series. Moreover, the S geometry of the SMA fibers produced greater changes in both length and diameter than the D-shaped geometry, as shown in Figure 3. For example, the length of the NT_D decreased from 30 to 28.8 mm, whereas the NT_S changed from 30 to 28 mm. In addition, the length and diameter of all SMA fibers changed significantly within 1 to 3 min of heat treatment. Based on this observation, a short-term heat treatment of only a few minutes could successfully activate the shape memory effect of cold-drawn SMA fibers. Therefore, in this study, the heat treatment period was determined to be 10 min during the pullout test.

### 3.2. Pullout Stress Versus Slip Response

**Figure 4:**
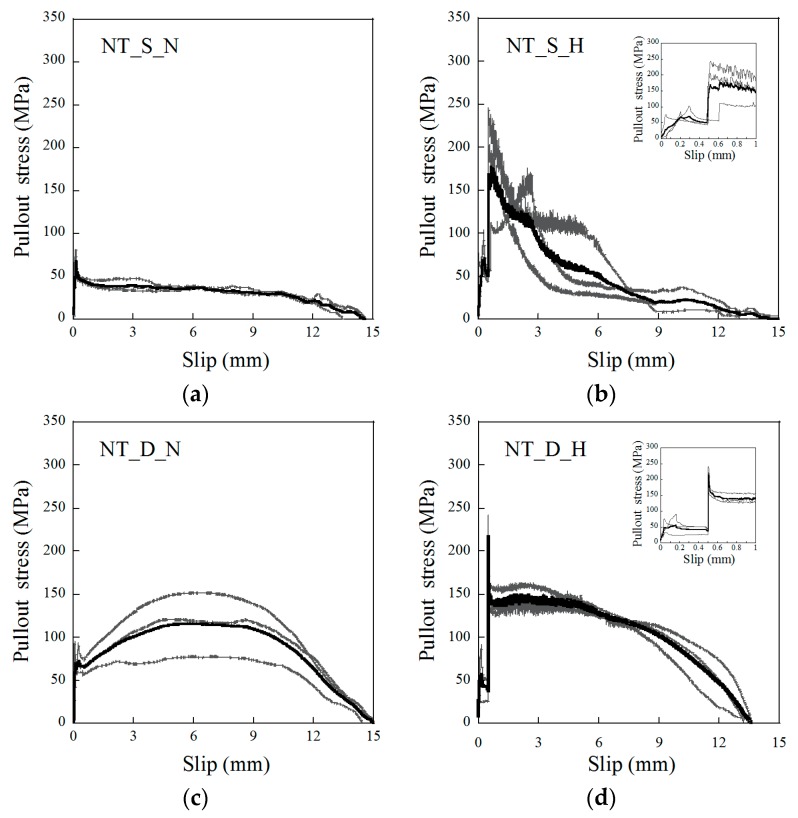
Pullout stress versus slip response of the NiTi fibers for (**a**) NT_S_N, (**b**) NT_S_H, (**c**) NT_D_N, and (**d**) NT_D_H. The insets in (**b**) and (**d**) are close-ups of the 0 to 1 mm slip regions for each graph.

**Figure 5:**
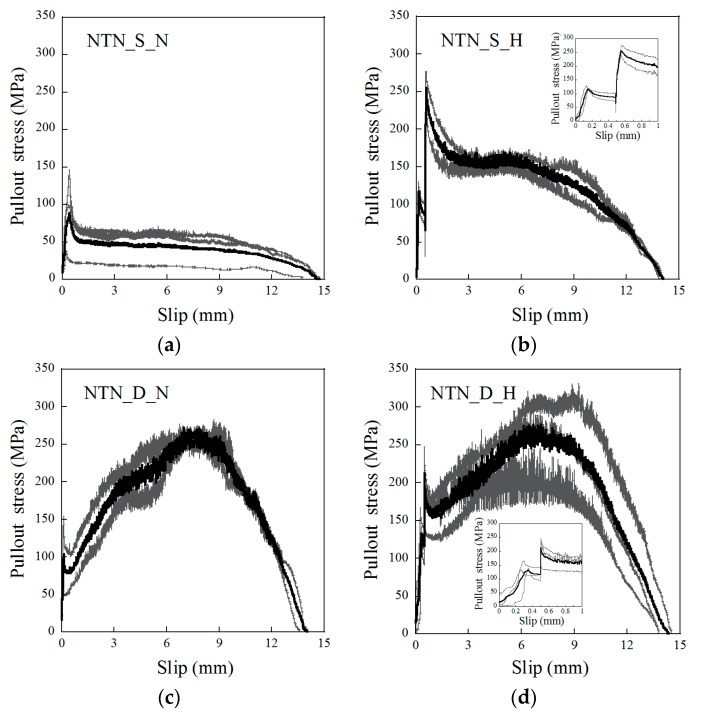
Pullout stress versus slip response of the NiTiNb fibers for (**a**) NTN_S_N, (**b**) NTN_S_H, (**c**) NTN_D_N, and (**d**) NTN_D_H. The insets in (**b**) and (**d**) are close-ups of the 0 to 1 mm slip regions for each graph.

The pullout stress versus slip curves of the NT fibers are provided in Figure 4, while those of the NTN fibers are shown in Figure 5. The pullout responses of the SMA fibers clearly changed relative to the different fiber alloys and geometries, and if the heat treatment was applied. The dog-bone-shaped SMA fibers largely produced a better pullout resistance than the smooth SMA fibers because the former utilized a mechanical interaction between the bulged end of the fiber and mortar matrix, unlike the smooth fibers, which only used the frictional bond resistance at the interface. In addition, for the short heat treatment samples, after the 0.5 mm initial fiber pullout, there was a noticeable increase in the pullout stress, even though the slip was a constant 0.5 mm, as shown in Figure 4b,d and Figure 5b,d. These figures additionally provide close-up graphs of the pullout response from the 0 to 1.0 mm slip. During the initial pullout to the 0.5 mm slip, the pullout stress reached its first peak point at a slip between 0.1 and 0.3 mm, and then decreased until the slip was 0.5 mm. When the slip reached 0.5 mm, it was kept constant for 10 min. For the test series with the heat treatment, the pullout stress at 0.5 mm slip immediately increased during the heat treatment above the initial peak stress. However, for the samples without a heat treatment, there was no change in the pullout stress at the 0.5 mm slip. Furthermore, among the dog-bone-shaped fibers, the NTN consistently showed a higher pullout resistance than the NT fibers, regardless of heat treatment. The higher pullout resistance of the NTN_D fibers over the NT_D fibers likely originated from the higher strength of the NTN.

Kim et al. [10] investigated the effects of heat treatment on the pullout resistance of SMA fibers embedded in the mortar matrix. They applied the pre-heat treatment (at 200 °C for 8 h) prior to the pullout test and then additional heat treatment (200 °C) was applied during the pullout test. The heat treatment of SMA fibers considerably recovered their reduced diameter after cold-drawing and consequently increased their pullout resistance. Moreover, Kim et al. [11] applied the pre-heat treatment (80 °C for 7 h) prior to the pullout test and then additional heat treatment (100 °C) during the pullout test. They reported that the heat treatment generally increased the pullout resistance regardless of the fiber compositions. Comprehensively, the enhanced pullout resistance of SMA fibers after heat treatment demonstrates their crack closing potentials in fiber-reinforced cementitious composites. However, current research requires not only a long time for pre-heat treatment of at least 7 h, but also continuous heat treatment during the pullout test. Therefore, it is necessary to reduce the time for heat treatment. Consequently, the pullout resistance was found to be enhanced by applying the heat treatment (300 °C) for only a short time (10 min) during the pullout test without pre-heat treatment.

### 3.3. Pullout Resistance during the Short Heat Treatment Period

**Figure 6:**
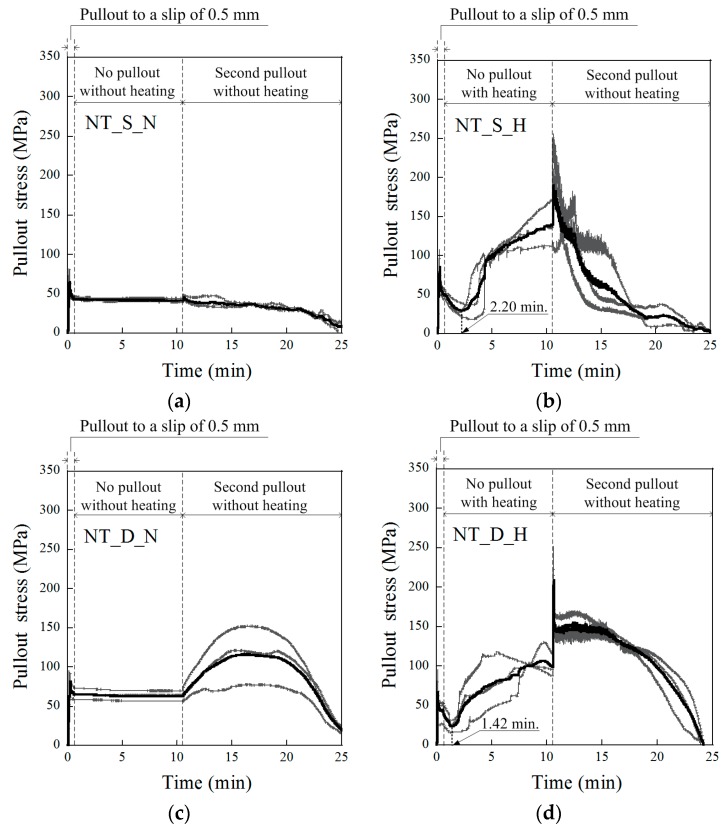
History of the pullout stress versus time for the NiTi fibers of (**a**) NT_S_N, (**b**) NT_S_H, (**c**) NT_D_N, and (**d**) NT_D_H.

**Figure 7:**
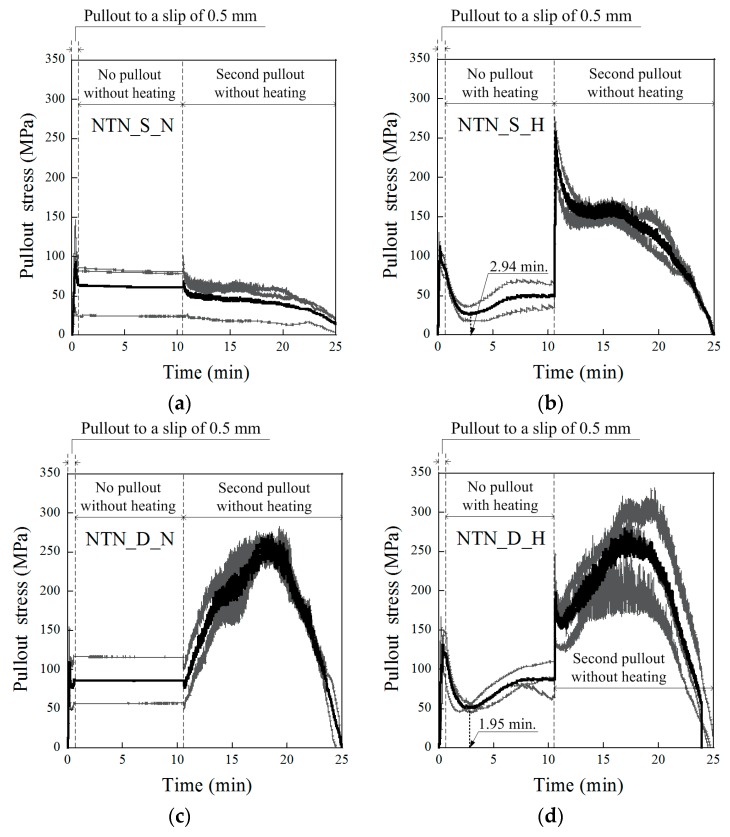
History of the pullout stress versus time for the NiTiNb fibers of (**a**) NTN_S_N, (**b**) NTN_S_H, (**c**) NTN_D_N, and (**d**) NTN_D_H.

**Figure 8:**
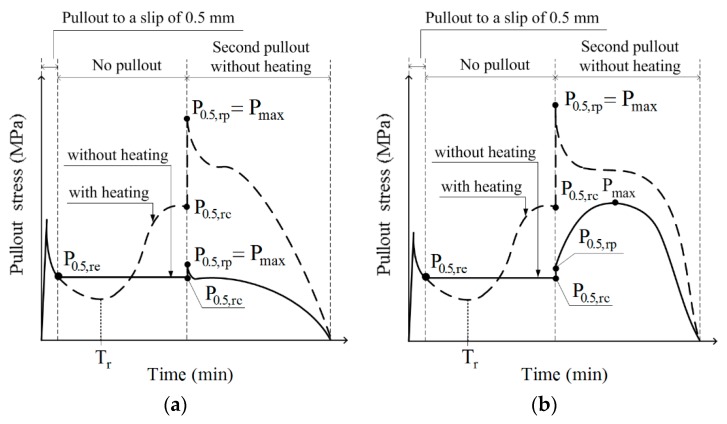

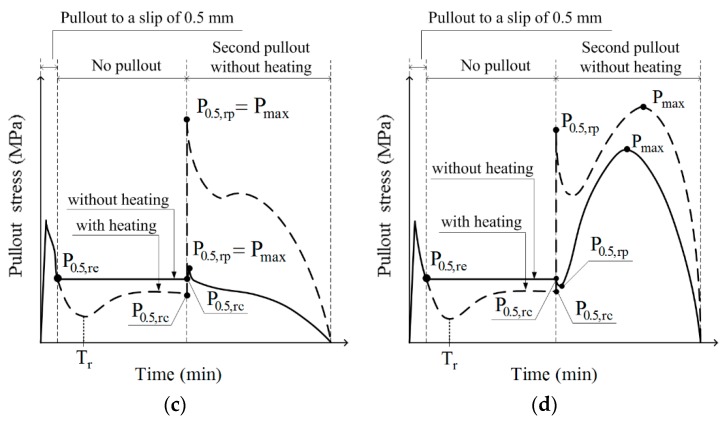
Typical pullout stress versus time curves of the shape memory alloy (SMA) fibers, showing the pullout parameters and pullout stress recovery for (**a**) NT_S, (**b**) NT_D, (**c**) NTN_S, and (**d**) NTN_D for samples with heating (solid lines) or without heating (dashed lines).

The changes in the SMA fiber pullout stress during the short heat treatment period are provided in Figure 6 and Figure 7 for the NT and NTN fibers, respectively. For the NT fibers, as shown in Figure 6a,c, if there was no heat treatment, the pullout stress was a constant value as the slip amount was kept at 0.5 mm for 10 min. However, for the series with the heat treatment, the pullout stress decreased at the beginning of the heat treatment, and then started to increase significantly, after 1–3 min, to a value greater than the residual pullout stress (P_0.5,re_) after 5 min of heating. After 10 min of heating, the pullout stress reached the recovered pullout stress (P_0.5,rc_), as illustrated in Figure 8, and the P_0.5,rc_ of the NT fibers was higher than the P_0.5,re_, regardless of the fiber geometry. When the fiber pullout was restarted after the 10 min heat treatment, the pullout stress of the fiber instantly increased to the re-pullout stress (P_0.5,rp_), as shown in Figure 6b,d and Figure 8. The P_0.5,rp_ was significantly higher than the P_0.5,rc_. The pullout resistance of the NT fibers initially decreased at the beginning of the heat treatment, owing to the thermal extension (or elongation) of the fibers. Then, the NT fibers began recovering the pullout resistance after 1–3 min, by activating their shape memory effects, i.e., shortening the length and expanding the diameter of the fibers; these eventually produced a higher P_0.5,rc_ than the P_0.5,re_. In Figure 8, the NTN fibers showed a similar response to the heat treatment as the NT fibers, with both maintaining a constant value for 10 min after the 0.5 mm initial slip when there was no heat treatment, whereas their values varied noticeably during the short heat treatment period. However, the P_0.5,rc_ of the NTN fibers was lower than their P_0.5,re_, unlike the NT fibers.

To quantitatively compare the pullout resistances of all test series, several parameters describing the SMA fiber pullout behavior are summarized in Table 4, including the P_0.5,re_, P_0.5,rc_, P_0.5,rp_, maximum pullout stress (P_0.5,max_), and pullout energy after the 0.5 mm slip to complete pullout (PE). After the short heat treatment time, the PE values were noticeably enhanced, as presented in Table 4, although the degree of enhancement varied according to the geometry and alloy of the fibers. The PE values of the dog-bone-shaped SMA fibers were clearly higher than those of the smooth SMA fibers: the value of NT_D_H was 1063.2 MPa-m, whereas that of NT_S_H was 525.5 MPa-m.

### 3.4. Pullout Stress Recovery Starting Time (T_r_) during the Heat Treatment

As observed in the pullout stress versus time curves of the SMA fibers during the short-term heat treatment in both Figure 6 and Figure 7, their pullout stress first decreased at the beginning of the heat treatment and then began increasing to the end of the heat treatment. The pullout stress recovery starting time was quite different based on the type of SMA fiber geometry and alloy. Table 5 provides the recovery starting times (T_r_) of the pullout stress relative to the test series during the short heat treatment period. The average T_r_ values of the test series (NT_S_H, NT_D_H, NTN_S_H, and NTN_D_H) were 2.20, 1.42, 2.94, and 1.95 min, respectively. The NT fibers usually produced faster T_r_ than the NTN fibers, while the D-shaped fibers produced faster T_r_ than the S fibers. The earlier pullout stress recovery of the dog-bone-shaped SMA fibers is thought to originate from their higher PE values, indicating a superior pullout resistance. Accordingly, the SMA fibers with a deformed geometry generating a larger PE are favorable for generating a faster crack closing ability. However, the parameters influencing the recovery start time (T_r_) require further investigation.

### 3.5. Pullout Stress Recovery Ratios

**Figure 9:**
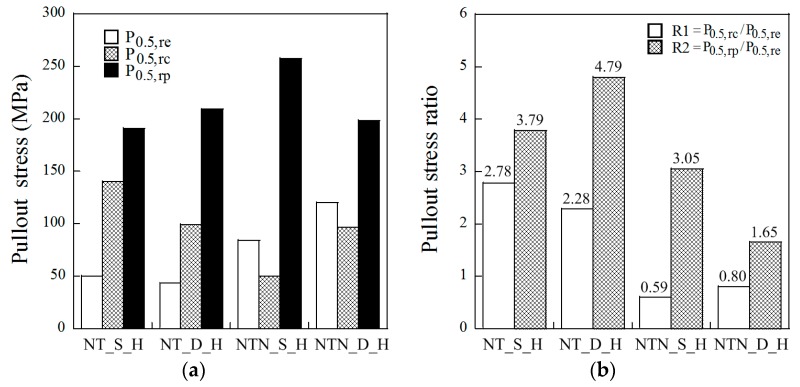
(**a**) Pullout stress and (**b**) pullout stress ratios owing to the heat treatment, for the different samples.

The ratios R_1_ and R_2_, between the pullout stresses, P_0.5,re_, P_0.5,rc_, and P_0.5,rp_, were calculated to quantify the crack closing ability of the SMA fibers during and after the short-term heat treatment. The ratio between the P_0.5,rc_ and P_0.5,re_ is denoted as R_1_, which represents the potential crack closing ability. The ratio between the P_0.5,rp_ and P_0.5,re_ was additionally analyzed and denoted as R_2_, which is thought to correlate with the ability to prevent crack reopening. Both R_1_ and R_2_ ratios are provided in Figure 9b. The NT fibers generally produce higher recovery ratios than the NTN fibers, as shown in Figure 9b; the values of R_1_ were 2.78, 2.28, 0.59, and 0.80 for NT_S_H, NT_D_H, NTN_S_H, and NTN_D_H, respectively, while those of R_2_ were 3.79, 4.79, 3.05, and 1.65 for NT_S_H, NT_D_H, NTN_S_H, and NTN_D_H, respectively. The R_1_ ratios of the NT fibers were higher than 1.0, whereas those of the NTN fibers were less than 1.0. Therefore, the pullout stress recovery during the short heat treatment period is higher for the NT fibers than the NTN fibers. Additionally, R_1_ values below 1.0 are not sufficient for closing cracks, whereas those higher than 1.0 are indicative of a satisfactory or strong crack closing capacity. The R_2_ ratios of the NT fibers were also higher than the NTN fibers. Therefore, the NT fibers after the heat treatment displayed a higher pullout resistance during the second pullout process.

### 3.6. Pullout Energy Ratios

**Figure 10:**
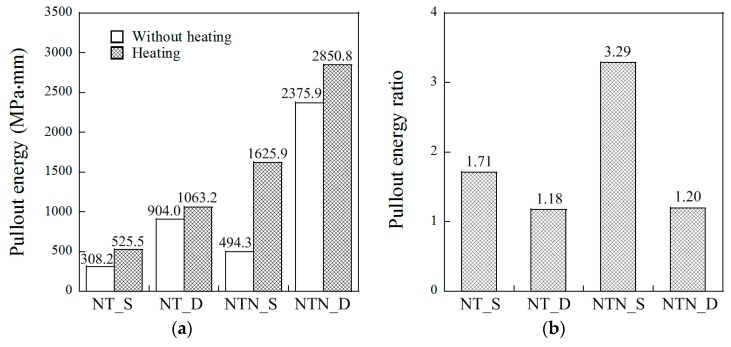
(**a**) Pullout energy (for samples with heating (grey) or without heating (white)), and (**b**) pullout energy ratio owing to the heating treatment.

The short heat treatment period also noticeably increased the amount of pullout energy after the 0.5 mm initial slip to complete fiber pullout, as provided in Table 4 and Figure 10a.

The enhanced SMA fiber pullout energy after the heat treatment originated from the lateral recovery of the fiber diameter due to shape memory effects. To quantitatively compare the heat treatment effect on the PE, the pullout energy ratios between samples with and without heat treatment were estimated and are shown in Figure 10. The pullout energy ratios of the NT_S, NT_D, NTN_S, and NTN_D were 1.71, 1.18, 3.29, and 1.20, respectively. The higher pullout energy ratio is thought to be favorable for resisting crack reopening. The NTN_S fiber produced the highest pullout energy ratio, while the NTN_D fiber produced the highest pullout energy. In addition, it was also evident that the smooth fibers generated higher pullout energy ratios than the dog-bone-shaped fibers because the pullout energy of the D fiber was much higher than that of the S fiber for the series without heat treatment. The *PE* value of NTN_S_N was 494.3 MPa-mm, while that of NTN_D_N was 2375 MPa-mm.

## 4. Conclusions

This study investigated the effects of applying a short heat treatment period (10 min) to shape memory alloy (SMA) fibers on their geometry and pullout resistance. The short heat treatment time clearly activated the shape memory effects, eventually generating the pullout stress recovery of SMA fibers in mortar. The conclusions below can be drawn from this experimental study:

- During the short heat treatment period, the length of the cold-drawn SMA fibers clearly decreased, whereas their diameter expanded due to the shape memory effect. The NiTi fibers generally showed greater shape memory effects in both diameter and length than the NiTiNb fibers, while the smooth geometry SMA fibers had greater shape memory effects than the dog-bone-shaped geometry fibers;
- SMA fibers with a dog-bone-shaped geometry generally showed higher pullout resistances than those with a smooth geometry;
- The short heat treatment period noticeably increased the pullout stress, although the degree of enhancement varied, relative to the SMA fiber alloy and geometry: (1) the NiTi fibers generally produced a faster pullout stress recovery than the NiTiNb fibers, while the SMA fibers with dog-bone-shaped geometries showed a faster recovery than those with a smooth geometry; (2) the NiTi fibers revealed higher pullout stress ratios during and after the heat treatment than the NiTiNb fibers; and (3) the SMA fibers with dog-bone-shaped geometries generated a larger amount of pullout energy (*PE*), which is favorable for faster crack closing.

Self-healing concrete can close (heal or fill) cracks; however it requires a few days minimum to heal the cracks. Consequently, in this study, we found that the SMA-FRCCs have a fast crack closing capacity (just only 10 min). The crack-closing behavior of SMA-FRCCs under a load is now under investigation and the parameters influencing the recovery start time require further investigation. Moreover, it is also necessary to investigate the residual stress of SMA-FRCCs [26].

## Figures and Tables

**Table 1:** Conditions of the single-fiber pullout tests.

Alloy	Fiber Diameter (mm) ^a^	Shape	Heat Treatment	Notation
NiTi	1.0 → 0.96	Smooth	No	NT_S_N
			Yes	NT_S_H
		Dog-bone shaped	No	NT_D_N
			Yes	NT_D_H
NiTiNb	1.12 → 1.08	Smooth	No	NTN_S_N
			Yes	NTN_S_H
		Dog-bone shaped	No	NTN_D_N
			Yes	NTN_D_H

^a^ → refers to the diameter change after the cold-drawing process.

**Table 2:** Matrix mixture composition by weight ratio and compressive strength.

Cement (Type 3)	Fly Ash	Silica Sand	High-Range Water-Reducing Admixture	Water	f′_ck_ (MPa)
1.00	0.15	1.00	0.009	0.35	55

**Table 3:** Properties of the shape memory alloy (SMA) fibers.

Notation	Length (mm)	Diameter (mm)	Young’s Modulus (GPa)	Tensile Strength (MPa)	Transformation Temperature (°C) ^a^			
					M_f_	M_s_	A_s_	A_f_
NT_S	30	0.96	41	973	0.7	36.7	43.2	62.8
NT_D								
NTN_S	30	1.08	21	1270	−	−100≥	−100≤	−
NTN_D								

^a^ M_f_ is the temperature at the end of martensitic transformation, M_s_ is the temperature at the start of martensitic transformation, A_s_ is the temperature at the start of austenitic transformation, and A_f_ is the temperature at the end of austenitic transformation.

**Table 4:** Pullout test results.

Notation	Sample	P_0.5,re_ (MPa)	P_0.5,rc_ (MPa)	P_0.5,rp_ (MPa)	P_max_ (MPa)	PE (MPa-mm)
NT_S_N	SP1	41.5	39.5	43.6	43.6	272.2
	SP2	46.2	44.2	48.8	48.8	335.2
	SP3	43.2	42.1	46.1	46.1	317.1
	**Aver. ^a^**	**43.7**	**41.9**	**45.7**	**45.7**	**308.2**
	STD ^b^	1.9	1.9	2.1	2.1	26.5
NT_S_H	SP1	56.6	112.5	181.9	181.9	471.3
	SP2	44.5	171.2	214.5	214.5	382.9
	SP3	49.9	136.1	254.9	254.9	658.0
	**Aver.**	**50.3**	**139.9**	**190.6**	**190.6**	**525.5**
	STD	4.9	24.1	29.9	29.9	114.7
NT_D_N	SP1	58.7	56.0	58.1	94.4	578.7
	SP2	73.5	69.1	47.9	152.3	1137.0
	SP3	64.2	64.2	65.8	121.2	959.1
	**Aver.**	**65.6**	**63.1**	**65.7**	**116.4**	**904.0**
	STD	6.4	5.4	7.3	23.7	232.9
NT_D_H	SP1	51.2	95.0	251.0	251.0	1123.9
	SP2	53.2	87.6	232.3	232.3	959.7
	SP3	26.3	115.2	193.7	193.7	1106.7
	**Aver.**	**43.6**	**99.3**	**208.8**	**208.8**	**1063.2**
	STD	12.2	11.7	23.9	23.9	73.7
NTN_S_N	SP1	23.9	23.9	26.3	26.3	181.5
	SP2	85.9	81.0	85.5	85.5	641.8
	SP3	81.6	78.3	99.8	99.8	652.3
	**Aver.**	**63.8**	**61.1**	**69.3**	**69.3**	**494.3**
	STD	28.3	26.3	31.8	31.8	219.5
NTN_S_H	SP1	73.1	33.7	239.4	239.4	1472.1
	SP2	95.9	66.4	276.6	276.6	1780.3
	**Aver.**	**84.5**	**50.0**	**257.5**	**257.5**	**1625.9**
	STD	11.4	16.4	18.6	18.6	154.1
NTN_D_N	SP1	57.2	57.1	57.4	273.8	2073.3
	SP2	115.8	115.8	116.3	282.2	2358.4
	**Aver.**	**84.8**	**84.5**	**86.7**	**271.8**	**2375.9**
	STD	29.3	29.4	29.5	4.2	142.6
NTN_D_H	SP1	148.0	63.8	247.0	296.1	2600.0
	SP2	92.8	109.9	154.7	330.4	2865.0
	SP3	120.9	115.1	236.2	269.0	2031.1
	**Aver.**	**120.3**	**96.6**	**198.3**	**280.1**	**2850.8**
	STD	22.5	23.1	41.2	25.1	347.9

^a^ Aver.: average values; ^b^ STD: standard deviation.

**Table 5:** Pullout stress recovery starting time during heating (T_r_).

Notation	Sample	T_r_ (min.)
NT_S_H	SP1	3.35
	SP2	1.93
	SP3	3.17
	**Average**	**2.20**
NT_D_H	SP1	1.47
	SP2	1.51
	SP3	1.42
	**Average**	**1.42**
NTN_S_H	SP1	4.20
	SP2	2.77
	**Average**	**2.94**
NTN_D_H	SP1	3.14
	SP2	1.93
	SP3	1.95
	**Average**	**1.95**
